# Polygenic, autosomal, and stable spirotetramat resistance in *Chrysoperla carnea* resulting in increased fitness

**DOI:** 10.1371/journal.pone.0310142

**Published:** 2024-09-10

**Authors:** Shoaib Hassan, Sarfraz Ali Shad

**Affiliations:** Department of Entomology, Faculty of Agricultural Sciences and Technology, Bahauddin Zakariya University, Multan, Pakistan; Kerman University of Medical Sciences, ISLAMIC REPUBLIC OF IRAN

## Abstract

Green lacewing, *Chrysoperla carnea* (Stephens) is a generalist predator used as a biological control agent in agro ecosystems. In order to use chemical and biological control in an integrated way, it is advantageous to know about natural enemy resistance response to a selected chemical. To determine *C*. *carnea* spirotetramat resistance potential, a population collected from the field was selected in the laboratory. Then we determined how spirotetramat resistance was inherited and how much it impacts the fitness of *C*. *carnea*. After eighteen selections with spirotetramat, the selected population (Spiro-Sel) of *C*. *carnea* had a 47-fold of resistance when compared to an UNSEL population. Inheritance results showed that spirotetramat resistance was inherited as an autosomal, incompletely dominant and polygenic trait. The values of effective dominance decreased from 0.87 (incomplete dominant) to 0.00 (complete recessive) as the concentration of spirotetramat increased from 625 mg/L to 10000 mg/L. The Spiro-Sel strain had no cross resistance to chlorfenapyr (1.10-fold), deltamethrin (1.26-fold) and chlorpyrifos (1.27-fold). After 7 generations without selection pressure resistance to all experimental insecticides in the Spiro-Sel strain was stable. Fitness data of the Spiro-Sel, Cross A, Cross B, UNSEL and susceptible strains of *C*. *carnea* showed that spirotetramat resistance increased the fitness of the selected green lacewing population. Life history parameters like fecundity, net reproductive rate, and relative fitness of the Spiro-Sel strain significantly increased when compared to the susceptible or unselected strains of *C*. *carnea*. These findings show that *C*. *carnea* is a perfect candidate for integrated pest management (IPM) programmes that combine biological control methods with selective pesticide applications to manage a variety of insect pests. Additionally, it would reduce the possibility of pests developing pesticide resistance despite repeated applications. It would be an excellent choice for widespread releases and be effective in most spray programs.

## 1. Introduction

Green lacewing, *Chrysoperla carnea* (Stephens) (Neuroptera: Chrysopidae) is a generalist predator of economic importance [1]. It is also known as the aphid lion [2]. Its importance in agriculture is due to the voracious feeding of its larvae on a number of insect pest hosts and resistance to many insecticides [3]. It has been mass reared in many countries, including Pakistan [4] and used as a biological control agent in many different crops [5–7], vegetables [8] and orchards [9]. In field studies, *C*. *carnea* effectively controlled cotton aphids and whiteflies in Saudi Arabia, achieving over 90% pest reduction in sweet pepper and squash [8]; In Portugal, reduced olive moth infestations in Portuguese olive groves through enhanced biodiversity [10]. Larvae are predacious while adults feed on nectar, honeydew and pollen [11]. *C*. *carnea* is one of the most important predator species due to its wide range of habitats, good searching ability, and ease of rearing in the laboratory [12]. Due to these characteristics, *C*. *carnea* is the most studied species among its genera.

Arthropod resistance to pesticide is a worldwide problem for both the public health and agriculture sector [13]. Pesticide resistance in insect pests continues to increase. Resistance to one or more insecticides has been documented among 600 species of insects and mites [14]. Resistance has been reported to most insecticide groups, including carbamates, organophosphates, pyrethroids, spinosyns, neonicotinoids, and diamides. Natural enemy resistance to insecticides has been reported in fewer cases than in insect pests. About 304 cases of resistance in 38 natural enemies were reported in 2016. Although resistance to pesticide in natural enemies has been increasing, it is still relatively lower than that in pest species [15]. The development of resistance to pesticides in insect pests is generally undesirable, whereas in biological control agents, such resistance can be advantageous as it ensures their survival in treated environments. However, consequences of resistance on the fitness of pests and natural enemies may vary. These resistant natural enemies can be used in integrated pest management (IPM) programs [16]. *C*. *carnea* is one of the most important natural enemies which have developed resistance to pesticides. About 162 cases of resistance to insecticides have been reported in *C*. *carnea* [14]. But resistance, genetics of resistance and fitness cost of insecticide resistance to spirotetramat has not been reported previously. The mode of action of spirotetramt is as; it inhibits the e activity of acetyl-CoA decarboxylase (ACC), the first enzyme of the fatty acid/lipid biosynthesis pathway. In plants, spirotetramat converts into enol form that actually inhibits the ACC activity [17]. Many studies have been reported on spirotetramat resistance, including resistance development in *Oxycarenus hyalinipennis* Costa [18], fitness effects on *Drosophila suzukii* stock [19] and its toxicity effects on *Drosophila melanogaster* Meigen [20]. However, there is no report of spirotetramat resistance and its fitness cost on this natural enemy.

An organism faces many environmental stresses when it adapts to a new environment. Similarly, insect pests face many risk hazards in insecticide contaminated environments. Resistance development in an insect pest to an insecticide is an adaption that can be accompanied by high energetic costs that reduce the fitness of the insect as compared to its unselected counterpart [21]. Knowledge about the fitness costs of insecticide resistance in insect pests is a useful tactic in pest management. In addition to this, the fitness of natural enemies resistant to insecticides is an important key point for their use as a biological control agent. Fitness costs of insecticide resistant strains of *C*. *carnea* are not well studied and few reports have been published. Previously, the fitness costs in emamectin benzoate and spinosad resistant strains of *C*. *carnea* have been reported [22, 23]. Further studies on fitness costs of a resistant strain of *C*. *carnea* are required for its better and effective evaluation and use in integrated pest management programs.

Biological control agents can not only survive in insecticide contaminated environments, these are also able to perform better predation and parasitism [16]. Therefore, this study was conducted to evaluate resistance development to spirotetramat in *C*. *carnea*. Resistance dominance and stability of insecticide resistance in the absence of insecticide exposure and the effects of spirotetramat resistance on biological parameters were also studied Furthermore, the cross resistance potential to new chemistry and conventional insecticides was determined.

## 2. Materials and methods

### 2.1 Population collection and rearing

*C*. *carnea* adults were collected with the help of an aerial net from fields of wheat in Multan (30° 11′ 44 N; 71° 28′ 31 E) and brought into the laboratory of the Pakistan Agriculture Research Council (PARC) sub-station Multan, Pakistan. These adults were reared in the laboratory in plastic rearing cages (23 × 38 × 38 cm) containing holes of 5cm on their lateral sides for aeration. A black glossy paper was placed in the roof in the cages for egg laying purposes as it resembles the shady surface of the underside of leaves. *C*. *carnea* adults laid eggs on these sheets. Black glossy sheets were replaced on alternate days and eggs were removed. Eggs of *C*. *carnea* were placed in cells (vertical hole) (4×3mm) of plastic Perspex plates along with diet [24] (frozen eggs of *Sitotroga cereallela* Oliver). The eggs of *S*. *cereallela* were kept in freezer at 0°C for an hour to freeze them. The eggs of *S*. *cereallela* were frozen to avoid hatching. The perspex plate has four counter parts, with two muslin cloth and two plastic sheets having the same dimension of holes as the central main Perspex plate. Second and third instar diet was administered at 2 day intervals on the muslin cloth so that larvae were not disturbed. Larval stages completed development in these plates and pupae formed. After pupal formation, these pupae were transferred into an adult cage of *C*. *carnea* and named the Field Pop. Another population of *C*. *carnea* named susceptible was also used in this study. It had been reared in the laboratory of PARC for more than ten years. Laboratory conditions were maintained at temperature 25 ± 2°C, relative humidity (RH) 60–65%, and photoperiod 14:10 h (light:dark) during the experiment [25, 26].

### 2.2 Insecticides

The formulated insecticides chlorfenapyr (Squadron 150SL, FMC United), chlorpyrifos (Lorsban 40EC, Dow Agro Sciences), deltamethrin (Decis Super 10EC, Bayer Crop Sciences, Pakistan) and spirotetramat (Movento 150OD, Bayer Crop Sciences, Pakistan) were used in this study.

### 2.3 Insecticide toxicity bioassay

Toxicity bioassays were conducted with a topical application method. Second instar larvae of *C*. *carnea* were exposed in the toxicity bioassay. Eggs of *C*. *carnea* were placed in the Perpex plate after collection from adult rearing cages. Five day old larvae in the second instar were collected from these Perpex plate [27]. Larvae were made inactive before insecticide exposure by keeping them in a freezer at 0°C for 30 seconds. These inactive larvae were kept in a petri dish (5cm diameter) and exposed to insecticide topically with a micro applicator (Burkard Manufacturing Co. Ltd., Hertfordshire, England). The micro applicator was calibrated to deliver a droplet size of 0.5μl of the solution on to the thorax of larva of *C*. *carnea*. Insecticide treated larvae were kept in transparent gelatin capsules along with their diet (frozen eggs of *S*. *cereallela*). Toxicity bioassays were conducted under the same laboratory conditions as mentioned above. Preliminary tests were run in the laboratory to determine the concentrations for initial bioassay that caused the mortality greater than 0 and lower than 100 to fit the Probit model. The concentrations used were 25ppm, 50ppm, 100ppm, 200ppm and 400ppm. However, the concentrations of the remaining bioassays varied between 78ppm to 20000ppm. Five treatments (i.e., concentrations of an insecticide) were made and each treatment was replicated 5 times. Five second instar larvae of *C*. *carnea* were exposed in each replication. A control treatment was treated only with water and replicated 5 times (5 larvae/replication). Thus a total 150 larvae were treated in each bioassay. Treatments were examined to calculate mortality 48 h after exposure to conventional insecticides and 72h after exposure to new chemistry insecticides [28]. Larvae of *C*. *carnea* were considered alive if they are moving or shifted from 2^nd^ instar to 3^rd^ instar.

### 2.4 Selection process

**Fig 1:**
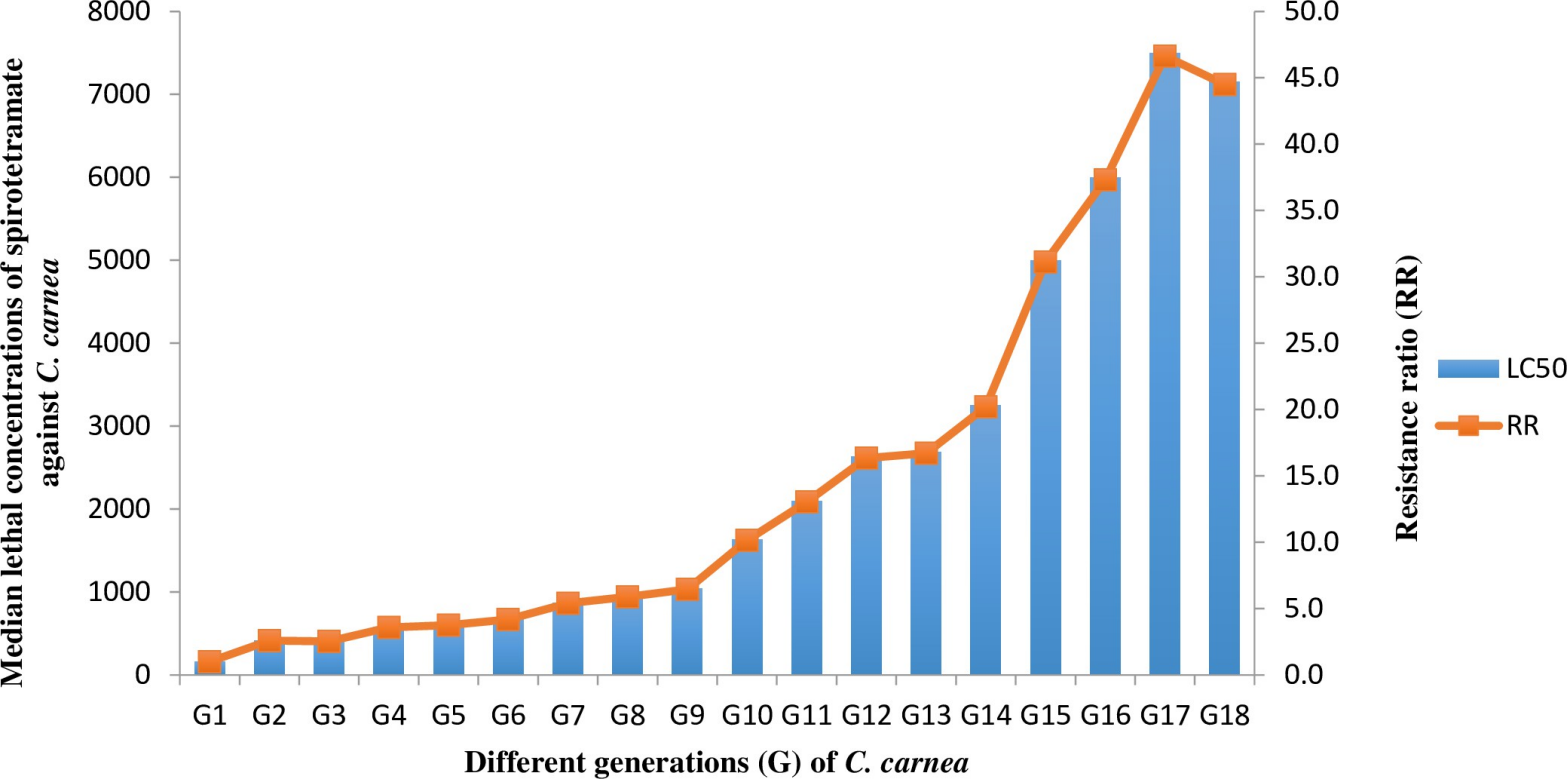
Selection history of spirotetramat in *Chrysoperla carnea*.

Field population of *C*. *carnea* was divided into two sub-populations. One sub population of *C*. *carnea* was further reared without exposure to any chemical for eighteen generations and named as UNSEL, while the 2^nd^ population was exposed to different concentrations of spirotetramat at every generation till 18^th^ generation and named as Spiro-Sel. Second instar larvae of *C*. *carnea* of each generation were continuously exposed to different concentrations of spirotetramat such as G_1_, G_2_, G_3_, G_4_, G_5_, G_6_, G_7_, G_8_, G_9_, G_10_, G_11_, G_12_, G_13_, G_14_, G_15_, G_16_, G_17_, to G_18_ to 200ppm, 300ppm, 400ppm, 500ppm, 600ppm, 700ppm, 800ppm, 900ppm, 1000ppm, 1100ppm, 1200ppm, 1500ppm, 2000ppm, 2500ppm, 3000ppm, 3500ppm, 5000ppm to 6000ppm, respectively. The insecticide exposure method was the same as in the toxicity bioassay but only one concentration was used against each generation. A total of 400 second instar larvae of *C*. *carnea* were treated in each selection. The concentrations of selection were made on the basis of number of surviving individuals. Selection history of the Spiro-Sel strain is given in Fig 1. Mortality data was taken after 72 h after treatment. Surviving larvae were reared for further generations.

### 2.5 Genetics of resistance to spirotetramat

Genetics of spirotetramat resistance in *C*. *carnea* was determined in the Spiro-Sel strain (G_17_) by making two reciprocal crosses. In first reciprocal cross, ten males of the Spiro-Sel strain were crossed with 10 females of the susceptible strain to get F_1,_ while in the second reciprocal crosss, ten females of the Spiro-Sel strain were crossed with 10 males of the susceptible strain to get F_1_’_._ To confirm the virginity of females, adults were separated within 24 h of eclosion. Male and female difference was determined by observing abdominal thickness. Abdomens were thick and longer in females and thin and shorter in males [29]. Individuals of the F_1_ were self-crossed to have a F_1_ Pool. Two backcrosses were produced: BC_1_ (Ten females of F_1_ × Ten males of susceptible) and BC_2_ (Ten females of F_1_ × Ten males of Spiro-Sel). These crosses provide enough offspring to conduct bioassays and determine lethal concentrations.

### 2.6 Dominance and effective dominance of spirotetramat resistance

Dominance of resistance (D_LC_) to spirotetramat in *C*. *carnea* was calculated with the following formula [30]:

$$D_{LC}=\frac{Log\;of\;{LC}_{50}\;of\;F1\;or\;F1^{\prime }\;or\;F1\;Pool-Log\;of\;{LC}_{50}\;of\;Susceptible}{Log\;of\;{LC}_{50}\;of\;Spiro\_Sel-Log\;of\;{LC}_{50}\;of\;Susceptible}$$

D_LC_ values change from 0 to 1, with D_LC_ = 0 showing completely recessive, D_LC_ = 1 showing completely dominant; while D_LC_ values 0 to 0.50 and 0.50 to 1, show an incompletely recessive and incompletely dominant nature of insecticide resistance, respectively.

The effective dominance (D_ML_) of the resistant strain of *C*. *carnea* due to exposure to spirotetramat was calculated through Bourguet, Genissel [31] method. Values of D_ML_ change from 0 to 1 (i.e. completely recessive to completely dominant). Mortality values of strains were used to determine D_ML_, as follows:

$$D_{ML}=(M\;F1–M\;susceptible)/(M\;Spiro‐Sel–M\;susceptible)$$

Where, M of Spiro-Sel, M F1 and M of susceptible are the mortality values used at specific insecticide concentrations for Spiro-Sel, F_1_ progeny and susceptible strains, respectively.

### 2.7 Inheritance pattern analyses

Chi-square test was used to test the hypothesis of monogenic type of resistance to estimate the number of genes responsible for resistance development. A null hypothesis tested that resistance was monogenic using the following equation [32]:

$$x^{2}=\frac{{\left(f-pn\right)}^{2}}{pqn}$$

At a particular dose of insecticide, f is mortality in BC_1_, p is the expected mortality at this dose and n is the numbers of individuals treated to a particular dose [33] while the q value is calculated as 1−p. The null hypothesis would be rejected when there is a significant difference (P<0.05) between fifty percent of observed and expected mortalities.

### 2.8 Cross-resistance evaluation

Cross-resistance (CR) in the Spiro-Sel strain of *C*. *carnea* was assessed with the exposure to other insecticides mentioned in the insecticide section as compared to that of the Field Pop of *C*. *carnea*. Cross-resistance ratio was calculated as:

CR=LC50ofinsecticidesinSpiro−Sel/LC50ofinsecticidesinFieldPop

### 2.9 Stability of spirotetramat resistance

Insecticide resistance stability was determined through rearing of the Spiro-Sel strain of *C*. *carnea* after 7 generations without insecticide exposure from G_18_ to G_25_. Toxicity bioassays of different insecticides were conducted at G_18_ and G_25_. Decrease of resistance (DR) to spirotetramat and other insecticides in the Spiro-Sel population was calculated by the following formula:

$$DR=\frac{Log\;of\;final\;LC50-Log\;of\;initial\;LC50}{n}$$

Where final and initial LC_50_’s was the LC_50’s_ of Spiro-Sel (G25) and Spiro-Sel (G18), respectively, and ‘n’ is the number of generations reared without insecticide exposure.

### 2.10 Study of fitness parameters

Life history traits of hybrids were assessed by making two reciprocal crosses: Cross A (Spiro-Sel ♀ × UNSEL ♂) and Cross B (Spiro-Sel ♂ × UNSEL ♀). In each cross, ten males and ten females were crossed to produce offspring of Cross A and Cross B. To study fitness parameters, ten pairs of adults of *C*. *carnea* were collected from the Spiro-Sel, UNSEL, susceptible, Cross A and Cross B population to record fecundity. Each treatment was replicated three times. Fecundity and adult lifespan were observed. First instar larvae (150) were separated randomly from each experimental population, Spiro-Sel, susceptible, UNSEL, Cross A and Cross B. Three replications were made, and 50 larvae were used in each replication. Weight of first instar larvae was recorded within 24h after hatching of eggs, and then reared in the capsules along with diet (frozen eggs of *S*. *cereallela*). All larval stages were reared in these capsules till pupation. Larval mortality, larval duration, larval weight of each instar, pupal duration, and pupal weight were recorded.

Eggs collection and counting was done daily until the death of females. Hatching percentage of eggs was calculated as:

$$Hatching\;(\%)=\frac{Total\;neonates}{Total\;eggs}\times 100$$

Net reproductive rate (R_0_) was calculated by following [34]:

$$R_{0}=\frac{N_{n+1}}{N_{n}}$$

In the above equation, N_n+1_ are the Total sum of offspring larvae and N_n_ denotes the number of parental populations.

Relative fitness was determined as given below [35]:

$$Relative\;fitness=\frac{R_{0}\;of\;any\;experimental\;strain}{R_{0}\;of\;UNSEL}$$

### 2.11 Statistical analysis

Mortality data was analyzed through probit analysis [36] with POLO Plus software [37] to determine the median lethal concentrations (LC_50_) with their 95% fiducial limits (FLs), slope with standard error (SE) and chi-square (χ^2^). Mortality in control treatment was corrected with Abbott [38] formula. If 95% FLs of LC_50_ values were overlapping, they were considered similar in toxicity [39]. At 5% level of significance, “General Linear Model” was used to analyze the data of fitness parameters of all tested strains by statistical software Statistix 8.1. Means were separated by using Least Significant Difference (LSD) test at P < 0.05 (5% probability level).

## 3. Results

### 3.1 Spirotetramat selection and its toxicity on different strains of *Chrysoperla carnea*

Spirotetramat selection on the Spiro-Sel strain of *C*. *carnea* for eighteen generations is given in Fig 1. *C*. *carnea* developed 44.5-fold of resistance to spirotetramat as compared to the Field strain after 18 generations. Toxicity of spirotetramat to the susceptible, Field Pop, Spiro-Sel (G_17_), F1, F1’, F1 Pool, BC1, BC2, UNSEL, Spiro-Sel (G_18_), Cross A, and Cross B strains is given in Table 1. There was a significant difference in resistance to spirotetramat in the susceptible, Field Pop and Spiro-Sel strains of *C*. *carnea* as their 95% FL values did not overlap. Furthermore, the Spiro-Sel (G_17_) strain developed 253 and 47-fold levels of spirotetramat resistance as compared to the susceptible and Field Pop, respectively. The resistance ratio of F1, F1’, F1 pool, BC1, and BC2 was 81, 69, 90, 69, and 100-fold, respectively, when compared to the susceptible strain, while it was only 15, 13, 17, 13, and 18-fold when compared to the LC_50_ of the Field Pop. In the fitness experiment, the resistance ratio of Spiro-Sel (G_18_), Cross A, and Cross B was 148, 99, and 114-fold, respectively, when compared to the UNSEL population.

**Table 1:** Toxicity of spirotetramat to various strains of *Chrysoperla carnea*.

**For inheritance study**								
**Strain**	**LC_50_ (95% FL) ppm**	**Slope± SE**	***χ* ^2^**	**Df**	**P**	**RR_1_^a^**	**RR_2_^b^**	**D_LC_^c^**
Susceptible	29.64 (20.81–43.42)	2.27 ± 0.45	1.08	4	0.71	1	-	
Field Pop	160.77 (98.69–340.31)	1.46 ± 0.39	0.17	4	0.49	5	1	
Spiro-Sel (G_17_)	7498.87 (4774.09–19351.32)	1.82 ± 0.48	1.18	4	0.87	253	47	
F1	2394.39 (1651.99–3445.79)	2.25 ± 0.45	2.53	4	0.64	81	15	0.79
F1’	2042.86 (1340.79–2981.49)	2.06 ± 0.43	2.60	4	0.63	69	13	0.76
F1 Pool	2670.99 (1796.65–4030.39)	2.02 ± 0.43	1.11	4	0.89	90	17	0.81
BC1	2042.97 (1312.51–3027.50)	1.96 ± 0.43	2.81	4	0.59	69	13	
BC2	2959.97 (2011.15–4524.54)	2.02 ± 0.43	1.70	4	0.79	100	18	
**For fitness study**								
**Strain**	**LC**_**50**_ **(95% FL) ppm**	**Slope± SE**	***χ*** ^**2**^	**Df**	**P**	**RR**_**1**_	**RR**_**3**_ ^**d**^	
UNSEL	48.31 (32.68–70.92)	2.11 ± 0.43	0.4	4	0.39	1.63	-	
Spiro-Sel (G_18_)	7156.30 (5102.47–10606.99)	2.36 ± 0.47	1.84	4	0.87	241.44	148.13	
Cross A	4788.79 (3304.02–6891.54)	2.25 ± 0.45	2.53	4	0.73	161.56	99.13	
Cross B	5536.50 (3858.37–8081.45)	2.24 ± 0.45	3.83	4	0.64	186.79	114.60	

^a^Resistance ratio = LC_50_ of tested strain / LC_50_ of susceptible strain

^b^Resistance ratio = LC_50_ of tested strain / LC_50_ of Field Pop

^c^Degree of dominance

^d^Resistance ratio = LC_50_ of tested strain / LC_50_ of UNSEL

F_1_ = Spiro-Sel ♂ × susceptible strain ♀

F_1_’ = Spiro-Sel ♀ × susceptible strain ♂

Cross A: Spiro-Sel ♀ × UNSEL ♂

Cross B: Spiro-Sel ♂ × UNSEL ♀

### 3.2 Toxicities of different insecticides to various strains of *Chrysoperla carnea*: Cross resistance and stability

Toxicities of spirotetramat, chlorfenapyr, deltamethrin, and chlorpyrifos to various strains of *C*. *carnea* are given in Table 2. Chlorpyrifos was significantly more toxic to the susceptible strain than three other tested insecticides (non-overlapping of 95% FL). The toxicities of spirotetramat, chlorfenapyr, and deltamethrin were similar as the 95% FL values of their LC_50_ overlapped. The resistance ratio of spirotetramat, chlorfenapyr, deltamethrin, and chlorpyrifos when compared to the susceptible strain of *C*. *carnea* was 5.42, 12.48, 1.78, and 1.68-fold, respectively, in the Field Pop and 1.63, 1.82, 1.60, and 2.20-fold in the UNSEL strain (G18).

**Table 2:** Toxicities of different insecticides to various strains of *Chrysoperla carnea*: Cross resistance and stability.

Strain	Insecticides	LC_50_ (ppm)	Slope± SE	*χ* ^2^	Df	P	RR	CR	DR
Susceptible	Spirotetramat	29.64 (20.81–43.42)	2.27 ± 0.45	1.08	4	0.71	1	-	-
	Chlorfenapyr	41.52 (29.29–57.59)	2.53 ± 0.49	1.2	4	0.43	1	-	-
	Deltamethrin	75.49 (49.37–114.42)	1.92 ± 0.41	0.91	4	0.32	1	-	-
	Chlorpyrifos	14.41 (9.36–20.23)	2.35 ± 0.49	0.68	4	0.33	1	-	-
Field Pop	Spirotetramat	160.77 (98.69–340.31)	1.46 ± 0.39	0.17	4	0.49	5.42	1	-
	Chlorfenapyr	518.32 (354.51–735.66)	1.61 ± 0.39	0.47	4	0.43	12.48	1	-
	Deltamethrin	134.71 (79.93–214.48)	1.64 ± 0.39	0.03	4	0.41	1.78	1	-
	Chlorpyrifos	24.21 (14.98–33.93)	2.36 ± 0.50	1.36	4	0.35	1.68	1	-
UNSEL (G18)	Spirotetramat	48.31 (32.68–70.92)	2.11 ± 0.43	0.4	4	0.39	1.63	-	-
	Chlorfenapyr	75.68 (52.47–105.26)	2.47 ± 0.48	0.95	4	0.38	1.82	-	-
	Deltamethrin	120.65 (72.59–184.48)	1.77 ±0.40	0.5	4	0.44	1.60	-	-
	Chlorpyrifos	31.65 (21.00–44.41)	2.35 ± 0.48	0.98	4	0.38	2.20	-	-
Spiro-Sel (G18)	Spirotetramat	7156.30 (5102.47–10606.99)	2.36 ± 0.47	1.84	4	0.87	241.44	44.51	-
	Chlorfenapyr	571.15 (384–937.47)	1.89 ± 0.42	0.04	4	0.57	13.76	1.10	-
	Deltamethrin	169.61 (116.10–251.82)	2.11 ± 0.43	0.23	4	0.38	2.25	1.26	-
	Chlorpyrifos	30.72 (21.49–41.49)	2.75 ± 0.53	0.94	4	0.39	2.13	1.27	-
Spiro-Sel (G25)	Spirotetramat	5536.50 (3858.37–8081.45)	2.24 ± 0.45	3.83	4	0.43	187.00	34.44	-0.02
	Chlorfenapyr	420.48 (295.77–602.14)	2.35 ± 0.46	3.1	4	0.54	14.00	0.81	-0.02
	Deltamethrin	156.80 (115.78–212.41)	2.94 ± 0.54	1.55	4	0.82	5.00	1.16	-0.02
	Chlorpyrifos	29.96 (20.02–41.36)	2.46 ± 0.49	0.95	4	0.92	1.00	1.24	-0.01

RR (Resistance ratio) = LC_50_ of tested strain / LC_50_ of susceptible strain

CR (Cross resistance ratio) = LC_50_ of tested strain / LC_50_ of Field Pop

DR = decrease in resistance

Cross-resistance results showed resistance to chlorfenapyr (1.10-fold), deltamethrin (1.26-fold), and chlorpyrifos (1.27-fold) did not increase significantly when compared to the Field Pop (overlapping of 95% FL values of LC_50_).

Resistance of the Spiro-Sel strain to all tested insecticides remained stable when reared without exposure to spirotetramat from generation 18 to 25 (Overlapping of 95% FL). The rate of resistance decline was -0.02, -0.02, -0.02, and -0.01, respectively, for spirotetramat, chlorfenapyr, deltamethrin, and chlorpyrifos.

### 3.3 Inheritance of spirotetramat resistance

**Fig 2:**
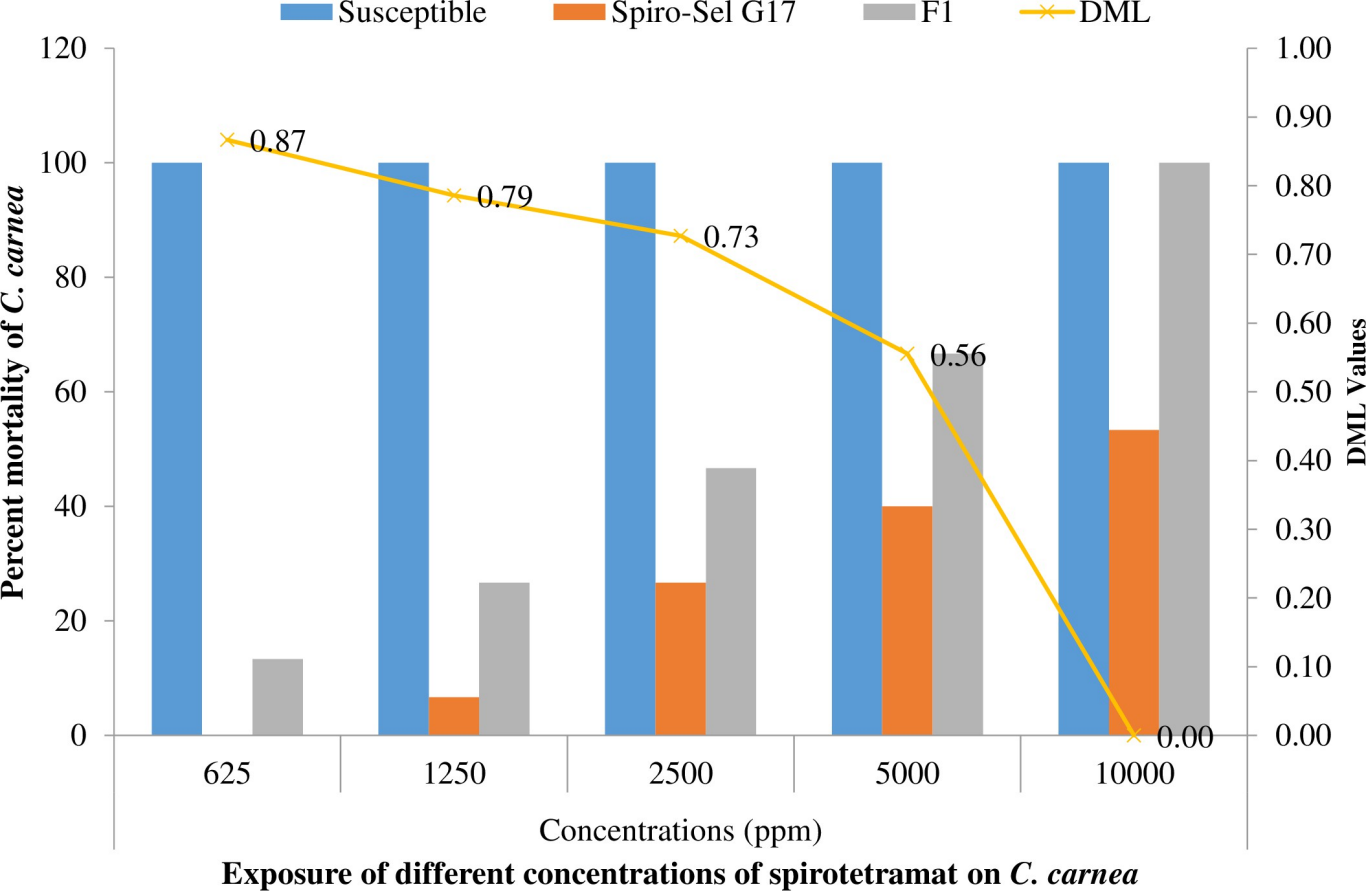
Effective dominance (D_ML_) of spirotetramat resistance in *Chrysoperla carnea*.

The overlapping 95% FL values of both reciprocal crosses confirm that spirotetramat resistance in the Spiro-Sel strain was inherited autosomally with no maternal effects. Dominance values (DLC) of F1, F1’, and F1 Pool were 0.79, 0.76, and 0.81, respectively, indicating that spirotetramat resistance in *C*. *carnea* was incompletely dominant (Table 1). The values of effective dominance (DML) decreased from 0.87 to 0.00 as the concentration of spirotetramat increased from 625 mg/L to 10000 mg/L (Fig 2). The DML values confirm that resistance to spirotetramat was completely recessive at the highest concentration of spirotetramat.

The monogenic model of spirotetramat inheritance revealed a polygenic mode of inheritance of spirotetramat resistance in *C*. *carnea* because at more than 50% of the studied doses, the expected and observed mortalities were significantly different (P<0.05) (Table 3).

**Table 3:** Monogenic model of inheritance for resistance to spirotetramat by comparing the observed and expected mortalities of BC_1_ of *Chrysoperla carnea*.

BC_1_					
Concentration(ppm)	n	Observed mortality	Expected mortality	*χ* ^2^ (df = 1)	p
625	15	0.20	0.10	1.25	0.263
1250	15	0.33	0.20	2.96	0.085
2500	15	0.53	0.37	7.08	0.008
5000	15	0.67	0.57	16.66	<0.001
10000	15	1.00	0.77	41.09	<0.001

P = Probability values were considered significantly different at P < 0.05.

### 3.4 Life-history parameters

Table 4 lists the means, standard errors, and statistical values of several life-history characteristics for the *C*. *carnea* Spiro-Sel, susceptible, UNSEL, Cross A, and Cross B strains. In comparison to the susceptible strain and UNSEL, the Spiro-Sel females produced significantly more eggs. The hatchability % and pupation rate of all tested strains were statistically similar. The numbers of progeny larvae of the Spiro-Sel strain were significant greater than that of all other studied strains, while both hybrid crosses had a statistically similar number of next generation larvae. The 1^st^, 2^nd^, and 3^rd^ instar larval weight of the Spiro-Sel strain was significant greater than that in both susceptible and UNSEL strains. Larval duration of Spiro-Sel was significantly different when compared to that of Cross A and susceptible strains but similar to that of Cross B and UNSEL strains. A significantly longer survival rate from first instar larvae to pupae was recorded in the Spiro-Sel strain as compared to all other studied *C*. *carnea* strains. The pupal weight of Spiro-Sel was significantly heavier than that of the susceptible and both hybrid crosses. There was a significantly shorter pupal duration in the Spiro-Sel strain as compared to the susceptible, UNSEL, Cross A, and Cross B strains. A comparison of the net reproductive rate of *C*. *carnea* strains showed a significant difference. The value of Ro for the Spiro-Sel, susceptible, UNSEL, Cross A, and Cross B was 10.11, 4.94, 6.41, 7.74, and 8.30, respectively. A highly significant difference was recorded in relative-fitness values of all experimental strains of *C*. *carnea*. The sequence of increase in relative fitness was susceptible< UNSEL< Cross A< Cross B< Spiro-Sel.

**Table 4:** Means of life history parameters of Spiro-Sel, susceptible, UNSEL, Cross A, and Cross B of *Chrysoperla carnea*.

Life history parameters	Spiro-Sel	Susceptible	UNSEL	Cross A	Cross B	Statistical values
No. of eggs laid per female	238.24 ± 2.5A	120.2 ± 1.1 D	144.87 ± 5.5 C	196.87 ± 1.8B	207.13 ± 5.65B	F = 159, P = 0.00
Hatchability %	84.89 ± 0.6	82.22 ± 3.2	88.67 ± 3.9	78.67 ± 4.8	80.15 ± 0.65	F = 1.60, P = 0.25
No. of Progeny larvae	202.22 ± 1.6A	98.83 ± 4D	128.13 ± 3.9C	154.80 ± 9.1B	166.07 ± 5.71B	F = 50.8, P<0.001
First instar weight (mg)	3.2 ± 0.2A	2.1 ± 0.1BC	2 ± 0.3C	2.6 ± 0.1B	2.3 ± 0.06BC	F = 7.80, P = 0.0040
Second instar weight (mg)	6.5 ± 0.2A	4.5 ± 0.3C	4.4 ± 0.2C	5.8 ± 0.1B	5.3 ± 0.33B	F = 13.9, P = 0.0004
Third instar weight (mg)	11.3 ± 0.4A	8.9 ± 0.1BC	8.8 ± 0.2C	9.8 ± 0.6B	10 ± 0.46B	F = 6.13, P = 0.0093
Larval duration (Days)	9.6 ± 0.1B	9.9 ± 0.1A	9.5 ± 0.1B	10 ± 0.1A	9.6 ± 0.07B	F = 6.67, P = 0.007
Survival rate (1st instar—pupae)	88.2 ± 1.2A	76.2 ± 2.1BC	78 ± 2.4B	71.6 ± 2.5C	73.6 ± 0.97BC	F = 11.3, P = 0.001
Pupal weight (mg)	15.3 ± 0.4A	13.9 ± 0.2C	14.8 ± 0.2AB	14.5 ± 0.0BC	14.6 ± 0.09B	F = 6.08, P = 0.0095
Pupal duration (days)	4.6 ± 0.1D	5.4 ± 0.1B	5.7 ± 0.1A	5.1 ± 0.0C	5 ± 0.03C	F = 32.5, P = 0.000
Pupation rate	91.5 ± 1.7	88.1 ± 1	88.7 ± 0.8	86.6 ± 0.5	87.4 ± 1.64	F = 2.44, P = 0.1155
Healthy adults	94.5 ± 0.8A	88.1 ± 1.3B	89.2 ± 1.1AB	86.2 ± 3.1B	84.1 ± 1.87B	F = 4.56, P = 0.0235
DT egg to adult (days)	16.5 ± 0.3B	18.3 ± 0.2A	18.3 ± 0.2A	17.8 ± 0.3A	18 ± 0.32A	F = 9.90, P = 0.0017
Net reproductive rate	10.11 ± 0.1A	4.94 ± 0.2D	6.41 ± 0.2C	7.74 ± 0.5B	8.30 ± 0.29B	F = 50.8, P = 0.046
Relative fitness	1.58 ± 0.04A	0.77 ±0.01 E	1 ± 0.0 D	1.21 ± 0.04C	1.30 ± 0.01B	F = 127, P<0.0001

## 4. Discussion

A systemic pesticide, spirotetramat, can actively travel through the xylem and phloem. It belongs to the Insecticide Resistance Action Committee’s Group 23 and is a chemical derivative of tetramic acid [13, 40]. By acting as an acetyl-CoA carboxylase inhibitor that prevents insect lipid production, it is effective against piercing-sucking insects [17, 41]. Additionally, spirotetramat has only somewhat negative and unwanted impacts on biological control agents of arthropods. Thus it is an excellent option for current IPM programs [40]. In our study, a Spiro-Sel strain of *C*. *carnea*, developed a 253-folds of resistance in comparison to the susceptible strain after continuous selection with spirotetramat for seventeen generations. Previously, *Phenacoccus solenopsis* Tinsley continuously treated for 13 generations which developed 328.69-fold resistance to spirotetramat when compared to the susceptible population [42]. The resistance development potential in Spiro-Sel strain of *C*. *carnea* may produce due to cytochrome P450 mono oxygenases as it plays a key role in detoxification. Previously it has been reported that high level of resistance in *Aphis gossypii* Glover to spirotetramat was present due to interaction of cytochrome P450 monooxygenase CYP380C6 [43]. Moreover, the resistance mechanism for spirotetramat resistance could be associated with selection pressure or population history and selection intensity or all of these. However, these results indicate the high potential of *C*. *carnea* to develop resistance to spirotetramat and the Spiro-Sel strain can be used effectively in pest management programs with this insecticide in an integrated way. Previously, *C*. *carnea* had been reported to develop resistance to carbaryl, acetamiprid, emamectin benzoate, buprofezin, and nitenpyram [22, 28, 44–46].

Pesticides either have positive or negative impacts on life-history features that are important for the ecological functions and reproduction of beneficial arthropods. Therefore, precise assessment of possible pesticide effects on biological control agents is essential for creating IPM tactics that work [22, 47]. In our study, selection with spirotetramat resulted in significantly higher relative fitness values in the Spiro-Sel strain and both hybrid crosses of *C*. *carnea* when compared to the UNSEL and susceptible strains. In comparison to the susceptible and UNSEL strain the pupal duration and developmental time from egg to adult of Spiro-Sel were significantly shorter while the number of eggs laid, net reproductive rate and percent hatchability increased. Previously, increased fitness of *C*. *carnea* was resulted from selection pressure with the insecticides spinosad [23], emamectin benzoate [22], profenofos, chlorpyrifos, lambda-cyhalthrin, alphamethrin, and deltamethrin [48]. Contrary to our findings, spirotetramat resistance resulted fitness costs in pests e.g., *P*. *solenopsis* and *O*. *hyalinipennis* [42, 49]. Moreover, in another study spiroteramate reduces the fitness of *D*. *suzukii* stock [19]. Based on these findings, the use of spirotetramat with *C*. *carnea* appears to be a promising option for controlling insect pests. Moreover, the high relative fitness value of the Spiro-Sel strain of *C*. *carnea* indicate that if spirotetramat usage continues, resistant strains reproduce and develop more rapidly than susceptible or UNSEL strains.

When using an insecticide in conjunction with biocontrol agents to manage a specific insect pest, it is crucial to understand the genetics of resistance inheritance [50, 51]. To better understand the mechanism of inheritance, effective dominance, degree of dominance, and number of genes promoting resistance development, it is useful to conduct genetic crosses between populations of natural enemies that are vulnerable to insecticides and those that are resistant to them [52–54]. Different modes of resistance inheritance occur among insect populations. How many generations were exposed to the insecticides and past histories of selection may affect inheritance [55]. The genetic characterization of spirotetramat resistance in *C*. *carnea* has not been the subject of any investigations that have been published. In this study, resistance of *C*. *carnea* to spirotetramat was inherited as an autosomal, polygenic, and incomplete dominant trait. Moreover, effective dominance results showed an inverse relation between the dominance pattern of spirotetramat resistance and concentration of insecticide. The degree of dominance may fluctuate when pesticide concentration is raised [56]. Our results on spirotetramat inheritance and effective dominance agreed with deltamethrin, pyriproxyfen, cyromazine, and acetamiprid resistance in *C*. *carnea* [26, 28, 57, 58]. Furthermore, Hu, Wang [59], Ijaz and Shad [60] and Ejaz, Ullah [54] also reported similar mode of spirotetramat resistance inheritance in *Panonychus citri* (McGregor), *O*. *hyalinipennis*, and *P*. *solenopsis*, respectively. In our study, the finding of partial dominant resistance coupled by many genes is an intriguing discovery. In an IPM program where different measures are taken to preserve existing populations of *C*. *carnea* in fields, the results of our study suggested that spirotetramat would be a compatible pesticide to employ in pest management programs.

The knowledge of resistance stability in the absence of pesticide exposure is critical for natural enemy usefulness in IPM programs [25]. Stable pesticide resistance in *C*. *carnea* is advantageous as it enables these predators to bear the harmful consequences of pesticides and guarantees their survival, particularly when insecticide selection pressure is reduced [44]. In our study, when the Spiro-Sel strain of *C*. *carnea* was reared without spirotetramat exposure, the resistance rate of decline to all experimental insecticides in Spiro-Sel was negligible, indicating that resistance remained stable. The reversion rate indicated that Spiro-Sel strain of *C*. *carnea* will lose 10-fold resistance after 12 generation without exposure to insecticide. The reversion to susceptibility may occur quickly in the absence of selection if resistance alleles imparted negative fitness. Resistance may last for extended lengths of time without being subject to selection pressure if the resistance development is coupled with positive fitness [25, 61]. The increased fitness of the Spiro-Sel strain might be possible reason of stable spirotetramat resistance. Our results on stability agree with previously reported buprofezin, spinosad, deltamethrin, pyriproxyfen, and nitenpyram stable resistance in *C*. *carnea* [26, 44, 58]. Unstable resistance to spirotetramat has has been reported in insect pests such as the citrus red mite [59], cotton mealybug [42], and dusky cotton bug [49]. The results on stability suggest that the Spiro-Sel strain could be effective in management of insect pests with spirotetramat applications in IPM programs as resistance to spirotetramat remained stable in *C*. *carnea* while unstable in the pest population.

The phenomenon of insecticide resistance in insects to unused pesticides from related or unrelated groups is known as cross-resistance [62]. Different pesticides may be used to control insect pests and lead to the development of cross resistance in pest populations that lessen the efficiency of pest management programs [63]. Furthermore, application of different pesticides may also induce cross resistance to these pesticides in natural enemies like green lacewing [28]. Therefore, the cross-resistance potential of spirotetramat resistant *C*. *carnea* populations to other insecticides could provide valuable information for effectively using the Spiro-Sel strain in IPM of insect pest. Cross-resistance can be induced by insecticidal target-site mutation, non-specific enzymes, and variables such as delayed cuticular penetration [64, 65]. When compared to the Field strain in this investigation, the Spiro-Sel strain of *C*. *carnea* displayed no cross-resistance to chlorfenapyr (1.10-fold), deltamethrin (1.26-fold), and chlorpyrifos (1.27-fold). The interesting and important thing is that susceptibility to none of the tested pesticides changed. It follows that selection with spirotetramat would not influence susceptibility if it did not result in cross-resistance to chlorfenapyr, chlorpyrifos, and deltamethrin. The current findings support earlier research which reported that a cyromazine-resistant strain of green lacewing did not possess cross-resistance to nitenpyram, cypermethrin, and chlorpyrifos [66]. Similarly, western flower thrips, *Frankliniella occidentalis* (Pergande), resistant to spirotetramat showed no cross resistance to formetanate, spinosad, acrinathrin, and methiocarb [67]. Contrary to our findings, spirotetramat resistant *Aphis gossypii* Glover possesed high cross resistance to bifenthrin and alpha-cypermethrin [68] and a spirotetramat resistant strain of *P*. *solenopsis* had a medium to high level of cross resistance to abamectin and bifenthrin [42]. Cross-resistance between different types of insecticides may develop due to their shared resistance development mechanism or other structural similarities [69]. Our cross resistance findings suggest that the tested insecticides cannot be integrated with *C*. *carnea* for management of insect pests.

In conclusion, this study sheds significant light on the stability, fitness, cross-resistance, effective dominance, and inheritance of spirotetramat resistance in the commonly used predator species, *C*. *carnea*. In IPM programs, mixing selective insecticides with biocontrol agents maximizes biological effectiveness and minimizes environmental repercussions. This study demonstrated that *C*. *carnea* can develop a high level of spirotetramat resistance that ensures its survival after continuous or repeated applications. Resistance to this tetramic acid derivative is inherited as polygenic and partially dominant traits. The development of resistance as an incompletely dominant trait may result in greater efficacy and long-term survival of this helpful predator. Furthermore, spirotetramat resistance resulted in improved fitness of the Spiro-Sel strain, demonstrating that resistant *C*. *carnea* works best with spirotetramat applications in the field to suppress resistant insect pests and avoid problems with secondary pest outbreaks or pest resurgence. The utility of spirotetramat in many IPM systems where biological control programs are used can be easily decided about its use. As in the current study, *C*. *carnea* has slight or no cross-resistance to chlorfenapyr, chlorpyrifos, and deltamethrin. Further field evaluation of spirotetramat resistant strain of *C*. *carnea* would be helpful before their use in IPM programs. Consequently, the widespread release of resistant green lacewings into cropping systems may be a useful strategy for the control of insect pests on a variety of crops.

## Supporting information

S1 Data (XLSX)
